# Pleural effusion adenosine deaminase: a candidate biomarker to discriminate between Gram-negative and Gram-positive bacterial infections of the pleural space

**DOI:** 10.6061/clinics/2016(05)05

**Published:** 2016-05

**Authors:** Ruolin Li, Junli Wang, Xinfeng Wang, Maoshui Wang

**Affiliations:** I First Affiliated Hospital of Guangxi Medical University, Department of Medicine Research, Nanning, Guangxi, China; II Affiliated Hospital of Youjiang Medical College for Nationalities, Center of Clinical Laboratory, Baise, Guangxi, China; III Shandong Provincial Chest Hospital, Department of Lab Medicine, Jinan, China

**Keywords:** Adenosine Deaminase, Gram-Negative Bacteria, Gram-Positive Bacteria, Treatment, Parapneumonic Pleural Effusion

## Abstract

**OBJECTIVES:**

Delay in the treatment of pleural infection may contribute to its high mortality. In this retrospective study, we aimed to evaluate the diagnostic accuracy of pleural adenosine deaminase in discrimination between Gram-negative and Gram-positive bacterial infections of the pleural space prior to selecting antibiotics.

**METHODS:**

A total of 76 patients were enrolled and grouped into subgroups according to Gram staining: 1) patients with Gram-negative bacterial infections, aged 53.2±18.6 years old, of whom 44.7% had empyemas and 2) patients with Gram-positive bacterial infections, aged 53.5±21.5 years old, of whom 63.1% had empyemas. The pleural effusion was sampled by thoracocentesis and then sent for adenosine deaminase testing, biochemical testing and microbiological culture. The Mann-Whitney U test was used to examine the differences in adenosine deaminase levels between the groups. Correlations between adenosine deaminase and specified variables were also quantified using Spearman’s correlation coefficient. Moreover, receiver operator characteristic analysis was performed to evaluate the diagnostic accuracy of pleural effusion adenosine deaminase.

**RESULTS:**

Mean pleural adenosine deaminase levels differed significantly between Gram-negative and Gram-positive bacterial infections of the pleural space (191.8±32.1 U/L *vs* 81.0±16.9 U/L, *p*<0.01). The area under the receiver operator characteristic curve was 0.689 (95% confidence interval: 0.570, 0.792, *p*<0.01) at the cutoff value of 86 U/L. Additionally, pleural adenosine deaminase had a sensitivity of 63.2% (46.0-78.2%); a specificity of 73.7% (56.9-86.6%); positive and negative likelihood ratios of 2.18 and 0.50, respectively; and positive and negative predictive values of 70.6% and 66.7%, respectively.

**CONCLUSIONS:**

Pleural effusion adenosine deaminase is a helpful alternative biomarker for early and quick discrimination of Gram-negative from Gram-positive bacterial infections of the pleural space, which is useful for the selection of antibiotics.

## INTRODUCTION

Pleural infections, defined as parapneumonic effusion (PPE) or empyema, are associated with considerable morbidity, mortality and health care resource use 1-4. Recently, the incidence of pleural infection has increased 5-6. In a study of pleural infection in hospitalized adults that was conducted in the United States, the frequency was 3.96 cases per 100000 in 1996 and 8.10 cases per 100000 in 2008 7. Delay in the treatment of this type of infection may contribute to its high mortality. Treating all patients who have suspected pleural effusion (PE) with antibiotics while waiting for microbiological results is not a good option because this practice increases antibiotic resistance. Therefore, from the standpoint of clinical utility, it is essential to develop a rapid test differentiating Gram-negative from Gram-positive bacterial infections of the pleural space.

Adenosine deaminase (ADA), an essential enzyme in purine metabolism, catalyzes the hydrolytic deamination of adenosine to form inosine 8. ADA is specifically secreted by T lymphocytes and macrophages during infections and participates in cell-mediated immunity 9. This enzyme is present in most tissues as well as in the serum and is also secreted into biological fluids during the cellular immune response against intracellular pathogens, but its levels can also be increased in other pathological processes 10-11. ADA was reported to be a useful biomarker in the diagnosis of tuberculous pleurisy and has been characterized in many reports 12-15. In particular, a meta-analysis showed that PE ADA had a sensitivity of 0.92 and a specificity of 0.90 in the diagnosis of tuberculous pleurisy 16. PE ADA levels are usually higher in tuberculous PE patients compared with other patients but may occasionally be elevated in certain other cases. For example, more than 40% of parapneumonic patients and half of patients with lymphomatous effusions exceed the cutoff set for pleural tuberculosis (TB) 17. Pleural ADA levels are also elevated in empyema, legionnaires’ disease, pleural brucellosis, lymphoma and *Mycoplasma pneumoniae* pneumonia 17-20. Moreover, various other factors are associated with pleural ADA, such as age 21, pleural protein levels 22, smoking 23, polymorphonuclear leukocyte counts 24, CD4+ cell numbers 25, and sunitinib use 26.

Pleural ADA was also reported to be a predictor of the outcomes of talc pleurodesis 27,28 and ulcerative colitis 29 and to be a biomarker for the diagnosis of acute scrub typhus 30 and pneumonia 31. In the present retrospective study, we aimed to evaluate the diagnostic role of pleural ADA in discrimination between Gram-negative and Gram-positive bacterial infections of the pleural space.

## MATERIALS AND METHODS

This was a retrospective observational study conducted from June 2006 through May 2012 in the Department of Laboratory Medicine, Shandong Provincial Chest Hospital. The study was approved by the medical ethics committee of this hospital. Written informed consent was not required because of the retrospective nature of the investigation. Patient records/information were anonymized and de-identified prior to analysis.

Patients who met the following criteria were enrolled: (1) pleural ADA had been assayed; (2) a positive PE culture had been obtained, and a single bacterial strain had been isolated; and (3) lymphoma and active TB were not present.

### Measurements

Approximately 10 mL of PE sample was obtained with a needle at the same time as thoracocentesis and was then sent for ADA testing, biochemical testing and microbiological culture. ADA activity was measured in the PE via a kinetic method employing xanthine oxidase/peroxidase 32 and an automated clinical chemistry analyzer using commercially available kits (Maker, Sichuan, China). The intra-assay and inter-assay coefficients of variability were ≤5% and ≤10%, respectively. The cutoff value of ADA for TB diagnosis was 30 U/L. The levels of total protein (biuret method), total bilirubin (vanadate oxidation method), glucose (hexokinase method), lactate dehydrogenase (lactate-to-pyruvate method) and amylase (CNPG3 method) in the PE were also assayed using a chemistry analyzer.

Additionally, microbiological cultures and susceptibility testing were performed using the Vitek (bioMerieux, Hazelwood, MO, USA) system.

### Statistical analysis

Statistical analysis was carried out using SPSS 17.0 software and MedCalc Version 8.0.1.0. Continuous variables are given as the mean±standard error of the mean (SEM). Non-parametric tests were used because the ADA data had a skewed distribution, as determined using the Kolmogorov-Smirnov test. Differences in ADA levels between groups were analyzed using the Mann-Whitney U test and correlations between ADA and specified variables were quantified using Spearman’s correlation coefficient.

Receiver operator characteristic (ROC) analysis was performed to evaluate the ability of PE ADA to discriminate between Gram-negative and Gram-positive bacterial infections of the pleural space. The cutoff point was determined as the value of the parameter that maximized the sum of the specificity and sensitivity. Positive and negative likelihood ratios were also determined. A two-tailed *p* value <0.05 was considered statistically significant.

## RESULTS

### Study population

The baseline characteristics of the patients are shown in Table 1. A total of 76 patients were enrolled into the study, including patients with PPEs (n=35) and empyemas (n=41). The patients then were grouped into the following subgroups: 1) patients with Gram-negative bacterial infections, aged 53.2±18.6 years old, 81.6% (31/38) of whom were male and 44.7% (17/38) had empyemas and 2) patients with Gram-positive bacterial infections, aged 53.5±21.5 years old, of whom 76.3% (29/38) were male and 63.1% (24/38) had empyemas.

### Description of pleural infections

Among the 76 cultures, 36 (47.4%) were positive for Gram-positive cocci and 40 (52.6%) were positive for Gram-negative bacteria. *Enterococcus pneumoniae* was the predominant Gram-positive coccus and *Pseudomonas pneumoniae* was the predominant Gram-negative bacterium. Of all Staphylococcus isolates, 8 (66.7%) were methicillin-resistant *Staphylococcus aureus* (MRSA). Moreover, 25% of Pseudomonas isolates were multi-drug resistant organisms (MDROs), and 1 Escherichia isolate and 1 Klebsiella isolate were extended-spectrum β-lactamase (ESBL) producers.

### Levels of ADA and other biomarkers

Mean pleural ADA levels differed significantly between Gram-negative and Gram-positive bacterial infections of the pleural space (mean±SEM, 191.8±32.1 U/L *vs* 81.0±16.9 U/L, *p*<0.01) (Table 1). The levels of total protein, total bilirubin, glucose, lactate dehydrogenase and amylase in the PE did not differ significantly between the infection types (all *p*>0.05).

There was a low negative correlation between pleural total bilirubin and pleural ADA (r=-0.266, *p*<0.05). There was also a moderate negative correlation between ADA and glucose (r=-0.357, *p*<0.01). Meanwhile, a high positive correlation was observed between ADA and lactate dehydrogenase in the PE (r=0.769, *p*<0.01).

### Diagnostic performance of pleural ADA

**Figure 1:**
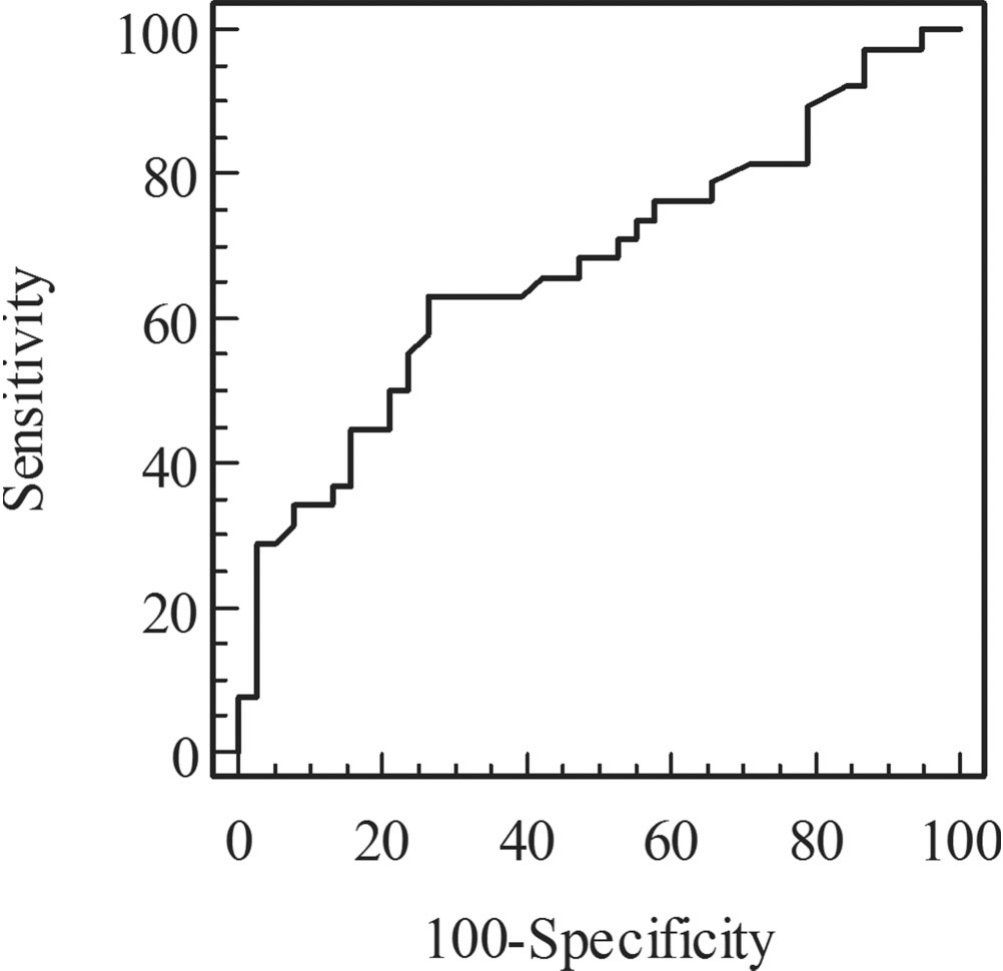
Receiver operator characteristic curve for pleural effusion adenosine deaminase to discriminate between Gram-negative and Gram-positive bacterial infections of the pleural space. The area under the curve was 0.689 (95% CI: 0.570, 0.792, *p*<0.01).

To evaluate the diagnostic performance of pleural ADA, ROC analysis was performed (Figure 1). The area under the ROC curve was 0.689 (95% confidence interval (CI): 0.570, 0.792, *p*<0.01) at the cutoff value of 86 U/L. Pleural ADA had a sensitivity of 63.2% (95% CI: 46.0-78.2%); a specificity of 73.7% (95% CI: 56.9-86.6%); positive and negative likelihood ratios of 2.18 and 0.50, respectively; and positive and negative predictive values of 70.6% and 66.7%, respectively.

## DISCUSSION

Rapid identification of the cause of pleural infection is defined by microbiological culture and effective antimicrobial therapy after diagnosis could potentially improve the outcome of pleural infection. However, in everyday clinical practice, 12-24 hours are usually required to obtain a Gram staining result once bacteria have been recovered from pleural cultures. Thus, an alternative method is needed for rapid diagnosis of the cause of PE. In the present study, we evaluated PE ADA for the detection and differentiation of Gram staining classification in pleural infection. In particular, the optimal cutoff value of PE ADA activity for discriminating between Gram-negative and Gram-positive bacterial infections of the pleural space was determined. The sensitivity and specificity of PE ADA were 63.2% and 73.7%, respectively. This biomarker is thus a possible alternative to conventional microbiological culture for discrimination of different types of pleural infection.

Currently, only a few studies have focused on discrimination between Gram-negative and Gram-positive bacterial infections. Charles PE et al. found that procalcitonin levels were markedly higher in patients with Gram-negative bacteremia than in those with Gram-positive bacteremia 33. This finding implied a diagnostic application in differentiating Gram-negative bacteremia from Gram-positive bacteremia. However, the results of that retrospective study could not be generalized to all patients with sepsis because only those with bacteremia were included. In another study, Xu XJ et al. 34 measured serum inflammatory cytokine levels in patients with septic shock using flow cytometry. The results showed that IL-6, IL-10, and TNF-α levels were significantly higher in patients with Gram-negative bacteremia than in those with Gram-positive bacteremia. Of the three cytokines, IL-10 was the most useful biomarker for Gram-negative bacteremia prediction in the derivation cohort and a cutoff value of 50 pg/mL showed a sensitivity of 70.8% and a specificity of 80.0%, with a positive predictive value of 89.5%.

Carroll NM et al. 35 developed a nested PCR protocol for detection of and discrimination between 14 species of Gram-positive and Gram-negative bacteria in samples of ocular fluids. In three culture-positive samples, complete concordance was observed between the molecular methods and culture results. PCR correctly identified the Gram staining classification of the organisms; however, high rates of false-positive nested PCR affected interpretation of the observed sensitivity of the test. Chan KY et al. 36 showed that the Gram-specific q-PCR test was highly specific and provided very good positive and negative predictive values for differentiating Gram-negative and Gram-positive systemic bacterial infections at the onset of clinical presentation in preterm infants. If the q-PCR test was positive, the chance of a true infection being present was 97.2%. However, only common Gram-negative and Gram-positive organisms were included in the genetic sequences of the primer/probes, so certain organisms could escape detection. The Hyplex BloodScreen PCR-ELISA system was also evaluated for direct identification of pathogenic bacteria in a large panel of positive BACTEC 9240 blood culture bottles 37. In contrast to conventional culture and biochemical identification techniques, which usually take 1-2 days, the Hyplex BloodScreen PCR-ELISA system generated results much more quickly. This method had a very high sensitivity, ranging from 96.6-100% for detecting various organisms. The specificity was also high, exceeding 97.5% for *Escherichia coli*. However, this method also requires microbiological culture.

Our current study differed from these previous studies in that ADA testing is routinely performed in clinical settings as a time-saving test. Although the diagnostic value of PE ADA was limited in discrimination between Gram-negative and Gram-positive bacterial infections of the pleural space, ADA measurement is available sooner than Gram staining results are and may possibly be an alternative to conventional microbiological culture for discrimination of pleural infections. The difference between pleural Gram-negative and Gram-positive bacterial infections in terms of the levels of PE ADA is related to immune responses upon stimulation with Gram-positive and Gram-negative bacteria, which may contribute to the role of ADA in lipopolysaccharide (LPS)-induced inflammatory responses. A previous study showed that IL-6, IL-10 and TNF-α levels were significantly higher in patients with Gram-negative bacteremia than in those with Gram-positive bacteremia 34. A recent study also found that inhibition of ADA attenuated the effect of LPS-induced inflammatory responses, such as by decreasing the levels of serum TNF-α, IL-1β, and IL-10. The results indicated that lowering ADA activity may be a novel and viable therapeutic approach to managing Gram-negative bacterial infection 38. In our study, the PE ADA level was lower in patients with Gram-negative bacterial infection compared with those with Gram-positive bacterial infection. This low ADA activity may be a protective factor against Gram-negative bacterial infection of the pleural space.

One of the limitations in this study was that no information was provided about the pleural cytology. In the study, approximately 60% of patients presented cell lysis during cytologic examination, and it has been reported that approximately 84% of purulent fluids contain lysed leukocytes 39. Previous studies have demonstrated a correlation between the pleural ADA level and CD4+ lymphocyte counts 40, suggesting that pleural cytology may have the ability to discriminate between pleural infections. Another limitation of the current study was its retrospective, non-randomized nature, but selection bias was likely limited because all patients with suspected pleural infection at our hospital undergo pleural culture and PE biochemical tests. When interpreting the results, the sample size and medium performance (sensitivity and specificity) should also be considered.

In conclusion, the levels of PE ADA in pleural infections caused by Gram-positive bacteria were high compared with the levels in pleural infections caused by Gram-negative bacteria. Subsequent analysis showed that PE ADA was a helpful alternative approach for early and quick discrimination of Gram-negative from Gram-positive bacterial infections of the pleural space, which would be useful for the selection of antibiotics. Because ADA measurement is available sooner than Gram staining results are, the former may possibly be an alternative to conventional microbiological culture for discrimination of pleural infections, particularly because drug susceptibility testing of clinical isolates has shown that antimicrobial resistance has become a serious concern in the treatment of pleural infection.

## AUTHOR CONTRIBUTIONS

Wang M and Wang X conceived this study. Li R collected the data. Li R and Wang J were involved in the analysis and interpretation of the data. Wang M wrote the manuscript. All authors read and approved the final version of the manuscript.

## Figures and Tables

**Table 1:** Patient characteristics.

	Gram-negative	Gram-positive
Age	53.2±18.6	53.5±21.5
Sex (Male, %)	30/36	30/40
Total protein (g/L)	31.7±4.2	39.4±2.3
Total bilirubin (mol/L)	19.6±4.4	14.7±4.4
Glucose (mmol/L)	3.54±1.29	3.04±0.86
Lactate dehydrogenase (U/L)	359.5±58.9	289.3±42.0
ADA (U/L)	44.2±10.8	83.2±21.2
Amylase (U/L)	29.5±13.3	42.0±16.7
